# Why Do Some Rainbow Trout Genotypes Grow Better With a Complete Plant-Based Diet? Transcriptomic and Physiological Analyses on Three Isogenic Lines

**DOI:** 10.3389/fphys.2021.732321

**Published:** 2021-09-01

**Authors:** Thérèse Callet, Mathilde Dupont-Nivet, Morgane Danion, Christine Burel, Marianne Cluzeaud, Anne Surget, Pierre Aguirre, Thierry Kerneis, Laurent Labbé, Stephane Panserat, Edwige Quillet, Inge Geurden, Sandrine Skiba-Cassy, Françoise Médale

**Affiliations:** ^1^INRAE, Université de Pau et des Pays de l'Adour, E2S UPPA, NUMEA, Saint-Pée-sur-Nivelle, France; ^2^Université Paris-Saclay, INRAE, AgroParisTech, GABI, Jouy-en-Josas, France; ^3^ANSES, Ploufragan-Plouzané Laboratory, Ploufragan, France; ^4^Pisciculture Expérimentale INRAE des Monts d'Arrée (PEIMA), Sizun, France

**Keywords:** aquaculture, genetic variability, LC-PUFA, plant-based diet, salmonid

## Abstract

Within the context of a growing aquaculture production coupled with a plateau of the production in the main components of aquafeeds (fish oil and fishmeal), recent studies have typically focused on replacing these feedstuffs with terrestrial plant ingredients for cultured carnivorous aquatic species, such as rainbow trout (*Oncorhynchus mykiss*). Substitution rates without adverse effects have, however, reached their limit. One potential way forward would be to take advantage of the genetic variability that exists in the salmonid population. However, to date, little is known about the underlying molecular mechanisms responsible for this genetic variability. The aim of the present research was to understand why some genotypes are better able to utilize plant-based diets devoid of marine resources. In this regard, three isogenic lines of rainbow trout (R23h, AB1h, and A22h), with similar growth when fed marine resources-based diets and which differ greatly in their responses to a plant-based diet, were fed with either a complete plant-based diet (V diet) or a marine resources-based diet (M diet) since first-feeding. Fish traits and the hepatic transcriptome of these three genotypes were compared after 5 months of feeding. First, differences in the ability to grow with the V diet observed amongst genotypes was not due to higher feed intake, but instead due to differences in feed efficiency. The comparison of transcriptome profiles revealed 575 (R23h vs. AB1h), 1,770 (R23h vs. A22h), and 2,973 (AB1h vs. A22h) probes differentially expressed amongst the three genotypes when fed the V diet. Interestingly, R23h and AB1h fish, which were the least affected by the V diet, exhibited the highest growth. These results demonstrate that these fish were able to maintain a high level of energy production and protein synthesis. Moreover, these genotypes were also able to activate pathways linked to lipid and cholesterol metabolisms, such as the biosynthesis of long-chain polyunsaturated fatty acids. Finally, as previously, immunity seems to also play an important role in the ability of fish to use the V diet, and further studies are needed to understand the mechanisms by which immunity interacts with growth.

## 1. Introduction

Fish have always been an important food commodity, providing human consumers with an important source of protein, essential n-3 long-chain polyunsaturated fatty acids (LC-PUFA), vitamins and minerals. A growing human population (Department of Economic and Social Affairs of the United Nations, 2013) combined with an increase in the average consumption of fish *per capita* have led to a greater demand for fish (FAO, 2016). Until the beginning of the 20th century, this demand was primarily provided by wild fish captures, while today, nearly half of the fish eaten are produced through aquaculture (FAO, 2016). This large growth in aquaculture production has led to an increasing demand for aquafeeds and thus for their main components, fish oil (FO) and fishmeal (FM). Because of their limited availability, FO and FM have been gradually substituted in fish diets by plant ingredients since 1990 (Tacon and Metian, 2008). This is particularly true for the diets for higher trophic level species, such as the rainbow trout (RBT).

The effects of FM and/or FO substitution by terrestrial plant ingredients have been extensively studied and the main obstacles to total substitution have been identified. First, plant-based diets are known to affect feed intake (Gomes et al., 1995; Espe et al., 2006; Dupont-Nivet et al., 2009), probably due to a change in diet palatability and/or due to a negative feedback induced by some plants components (Geurden et al., 2005). Second, fish fed highly substituted diets are often less efficient. The lower feed efficiency could stem from a lower nutrient digestibility and absorption, due to the presence of anti-nutritional factors (ANF), brought in by plant ingredients, impacting both the structure and the functioning of fish digestive tracts (Krogdahl et al., 2010). Nutrient deficiencies or inadequacies, such as the lack of LC-PUFA and cholesterol, a lower level of phospholipids, the presence of indigestible carbohydrates, or an unbalanced amino acid profile in plant-based diets, impair metabolism, particularly in the liver (Leaver et al., 2008; Panserat et al., 2008, 2009; Geay et al., 2011; Morais et al., 2011; Tacchi et al., 2012; Xue et al., 2015). Among the most severely impacted pathways are the LC-PUFA and cholesterol biosynthesis pathways (Leaver et al., 2008; Panserat et al., 2008; Morais et al., 2011; Tacchi et al., 2012). The protein biosynthesis capacity is decreased and a modification in the amino acid metabolism is observed when FM is replaced by vegetable meals (Panserat et al., 2008; Tacchi et al., 2012; Xue et al., 2015). The total replacement of both FM and FO by plant-based ingredients in salmonid feeds is also associated with changes in carbohydrate metabolism (Panserat et al., 2009; Geay et al., 2011). Finally, plant-based diets are also known to affect fish immunity parameters and the expression of genes involved in immunity is also altered when FO and/or FM are highly replaced by plant ingredients (Leaver et al., 2008; Panserat et al., 2008; Geay et al., 2011; Tacchi et al., 2012). Thanks to these research efforts, the proportion of vegetable oils and vegetable meals have progressively increased in aquafeeds. Nevertheless, even though feed formulation has improved over the years, the complete substitution of both FO and FM from the first feeding with terrestrial plant ingredients is not possible today in RBT without impairing growth and survival (Le Boucher et al., 2012; Callet et al., 2017).

In addition, a genetic variability for the capacity to grow with plant-based diets has been demonstrated in the RBT population (Le Boucher et al., 2012). Some genotypes are thus better able to survive and grow than others, when fed such diets (Le Boucher et al., 2012; Callet et al., 2017). For salmonids, a study which focused on RBT analyzed two families with different growth capacities when fed a diet devoid of fishmeal, but only revealed a few markers, which were all linked to the immune response (Overturf et al., 2012). Finally, a recent study found several potential markers linked to feed intake and sensory perception among three different RBT isogenic lines (Callet et al., 2018). This last result suggests that differences in diet acceptance could explain the important variability observed in survival and growth during the first weeks after the first-feeding with plant-based diets (Callet et al., 2018). Ultimately, only a few studies have investigated this question and the mechanisms which would explain this genetic variability have yet to be identified.

The present study thus sought to understand why some genotypes are better able to utilize complete plant-based diets as this information could be used to further reduce the use of FO and FM. For this purpose and in order to increase the statistical power to detect differences, we used a specific animal material: three isogenic lines, namely R23h, AB1h, and A22h, known to have similar growth when fed a marine resources-based diet but divergent growth when fed a plant-based diet (Dupont-Nivet et al., 2009; Geurden et al., 2013; Callet et al., 2018). Differences between these three lines could result from differences in feed intake and/or differences in their capacity to efficiently utilize such diets. To identify and separate genes from metabolic and physiological pathways which are linked to differences in feed intake from those linked to differences in the capacity to use nutrients from a plant-based diet, hepatic transcriptomic profiles were therefore compared between these three lines either fed a marine resources-based diet *ad libitum*, a marine resources-based diet distributed as a restricted ration or a 100% plant-based diet *ad libitum*.

## 2. Materials and Methods

### 2.1. Experimental Diets

The two experimental diets, formulated to be isoproteic, isolipidic, and isoenergetic, were produced in the INRAE facility (UMR Numéa, Donzacq, France). Formulation, composition, fatty acid (FA) and amino acid profiles for both experimental diets are presented in Tables 1, 2. The marine resources-based diet (M diet) contained FM, FO and whole wheat as major ingredients. The complete plant-based diet (V diet) contained only plant products and was devoid of marine products. Fava bean, corn, soybean, wheat gluten, peas, and white lupin were used as vegetable meals and rapeseed, linseed and palm oils were used as vegetable oils. To meet the known nutritional requirements of rainbow trout, the V diet was supplemented with L-Lysine and L-methionine. An attractant mix was also added to the V diet.

**Table 1:** Ingredients and proximal composition of the two experimental diets: the complete plant-based diet (V diet) and the marine resources-based diet (M diet).

	**V diet**	**M diet**
**INGREDIENTS (%)**		
**Fishmeal** (Southern hemisphere, Sopropêche, France)	0	65
Extruded whole wheat (SudOuest Aliment, France)	4	21
Fava bean (CP)	10	0
Corn gluten (CP 60; Inzo, France)	17	0
Wheat gluten (CP 70; Roquette, France)	17	0
Soybean meal (CP 48; Inzo, France)	12	0
White lupin seed meal (Terrena, France)	5	0
Extruded peas (Aquatex, Sotexpro, France)	12.5	0
**Fish oil** (Southern hemisphere, Sopropêche, France)	0	11
Rapeseed oil (Daudruy, France)	6	0
Linseed oil (Daudruy, France)	3.6	0
Palm oil (Daudruy, France)	2.4	0
Soy-lecithin (Louis François, France)	2	0
L-Lysine (Eurolysine)	0.5	0
L-Methionine (Evonik, Germany)	0.5	0
CaHPO4.2H20 (18% P; 22% Ca)	3	0
Min., and Vit. premix, INRA^*a*^	3	3
Attractant mix^*b*^	1.5	0
**Composition (% DM)**		
Dry matter	96.9	97.6
Crude protein	51.4	50.1
Crude fat	18.5	19.4
Starch	9.6	14.1
Ash	6.5	12.7
Energy (kJ/g DM)	23.6	22.6

*DM, dry matter*.

a *Mineral premix (g or mg kg-1 diet): calcium carbonate (40% Ca), 2.15 g; magnesium oxide (60% Mg), 1.24 g; ferric citrate, 0.2 g; potassium iodide (75% I), 0.4 mg; zinc sulfate (36% Zn), 0.4 g; copper sulfate (25% Cu), 0.3 g; manganese sulfate (33% Mn), 0.3 g; dibasic calcium phosphate (20%Ca, 18%P), 5 g; cobalt sulfate, 2 mg; sodium selenite (30% Se), 3 mg; KCl, 0.9 g; NaCl, 0.4 g (UPAE, INRA); And Vitamin premix (IU or mg kg-1 diet): DL-a tocopherol acetate, 60 IU; sodium menadione bisulphate, 5 mg; retinyl acetate, 15,000 IU; DL-cholecalciferol, 3,000 IU; thiamin, 15 mg; riboflavin, 30 mg; pyridoxine, 15 mg; B12, 0.05 mg; nicotinic acid, 175 mg; folic acid, 500 mg; inositol, 1,000 mg; biotin, 2.5 mg; calcium pantothenate, 50 mg; choline chloride, 2,000 mg (UPAE, INRA)*.

b *Attractant mix: glucosamine, 0.5 g; taurine, 0.3 g; betaine, 0.3 g; glycine, 0.2 g; alanine, 0.2 g*.

**Table 2:** Fatty acids and amino acids profiles of the two experimental diets: the complete plant-based diet (V diet) and the marine resources-based diet (M diet) (2 mm) (DM, dry matter).

	**V diet**	**M diet**
**FATTY ACID COMPOSITION (% OF TOTAL FATTY ACID)**		
Saturated	19.8	39.2
MUFA	39.0	29.5
n-6 PUFA	24.0	4.7
18:2 n-6 (LA)	24.0	3.2
n-3 PUFA	17.1	19.1
18:3 n-3 (ALA)	17.1	1.1
20:5 n-3 (EPA)	0.0	9.5
22:6 n-3 (DHA)	0.0	5.2
**AMINO ACID COMPOSITION (% DM)**		
Aspartic acid (Asp)	3.5	3.9
Glutamic acid (Glu)	11.2	6.3
Alanine (Ala)	2.4	2.8
Arginine (Arg)	2.7	2.6
Cysteine (Cys)	0.7	0.4
Glycine (Gly)	1.8	2.8
Histidine (His)	1.0	1.0
Isoleucine (Ile)	2.0	2.0
Leucine (Leu)	4.3	3.3
Lysine (Lys)	2.2	3.3
Methionine (Met)	1.2	1.2
Phenylalanine (Phe)	2.4	1.9
Proline (Pro)	3.4	1.7
Serine (Ser)	2.1	1.7
Threonine (Thr)	1.5	1.9
Tyrosine (Tyr)	1.6	1.2
Valine (Val)	2.2	2.4

The V diet was devoid of eicosapentaenoic acid (EPA, 20.5 n-3) and docosahexaenoic acid (DHA, 22.6 n-3). It was lower in saturated FA and higher in MUFA, linoleic acid (LA, 18:2 n-6) and α-linolenic acid (ALA, 18:3 n-3) than the M diet. The two diets also differ in amino acid profiles, especially in glutamic acid content which was two times higher in the V diet than in the M diet. Both diets were extruded. Pellet size was changed during the experiment to adapt to fish body size (from 0.5 to 1.9 mm), but composition remained unchanged.

### 2.2. Animal Material

The three lines studied were three heterozygous isogenic lines denominated R23h, AB1h, and A22h and were produced in the experimental PEIMA facility (INRAE, Sizun, France). Homozygous lines were obtained after two generations of gynogenesis and further maintained by within line pair-mating (Quillet et al., 2007). In order to have enough fish for the entire experiment, all the oocytes were collected from 33 females from the B27 isogenic line (thus sharing the same genome), were mixed and then randomly divided in three batches. Each batches were then fertilizated with milts from an individual male from three other homozygous isogenic lines, namely R23, AB1, and A22 (Supplementary Figure 1). The differences observed between lines could then be attributed only to paternal effects. These lines were used for scientific research only. As fish share the same genotype within each line, the three isogenic lines are appropriate biological materials to work on, as a low phenotypic variability is expected within line.

### 2.3. The 5-Month Growth Trial

The experiment was performed in the experimental PEIMA facilities (Sizun, France). Before the first feeding, approximatively 330 fry from the three lines were randomly distributed into 27 different tanks (0.25 m^3^, 9 tanks per isogenic line). At this stage, no difference in fry mean body mass was detected (0.05 g). The density was adjusted in the course of the experiment. Tank water volume was gradually increased from 0.08 to 0.20 m^3^. Water was kept at a constant temperature of 11.4°C.

From first feeding [47 days post fertilization (dpf)], fish were fed *ad libitum* with either the M diet (*n* = 6 tanks/line) or the V diet (*n* = 3 tanks/line). Five weeks after the first feeding (75 dpf), fish from 3 of the 6 tanks fed the M diet *ad libitum* were fed a restricted ration (MR dietary treatment) (*n* = 3 tanks/line). Briefly, the quantity of feed refused by fish fed the V diet was estimated daily. The ration given to the MR group were updated every day in order to obtain fish which had a similar feed intake as those fed the V diet. Feed was continuously distributed with automatic feeders during the lighting period (8 h).

To monitor growth, two or three groups of 50 fish randomly sampled in each tank were weighed on different dates (88, 102, 116, 137, 151, and 189 dpf). Between 137 and 189 dpf (52-day trial), uneaten pellets were collected daily after each distribution and weighed. An estimation of uneaten and consumed quantities allowed for the estimation of the daily feed intake (DFI) and feed efficiency (FE):

(1) $$\begin{array}{l} DFI=\frac{NI}{{\left(BW_{f}^{0.8}\times BW_{i}^{0.8}\right)}^{0.5}\times 52} \end{array}$$

(2) $$\begin{array}{l} FE=\frac{BW_{f}-BW_{i}}{NI} \end{array}$$

with BW_*i*_ and BW_*f*_, the mean body mass (in g wet weight) at the beginning (137 dpf) and at the end (189 dpf) of the period, and NI the net feed intake [in g dry matter (DM)].

Mortality, if any, was recorded daily for each tank during the experiment, and the survival rate was estimated throughout the experiment.

### 2.4. Sampling

At 194 dpf, marking the end of the experiment, different tissues were collected 8 h after the last meal. First, 162 fish (*n* = 18 fish/condition) were randomly sampled, anesthetized, weighed, then euthanized with benzocaine in excess (bath at 60 mg.L^1^). For 81 fish (*n* = 9 fish/conditions), blood was removed from the caudal vein into heparinized syringes and centrifuged at 3,000 g for 5 min in order to measure plasmatic levels of glucose, triglycerides and cholesterol. The recovered plasma was immediately frozen and kept at −20°C. Livers were also sampled and were immediately frozen in liquid nitrogen and stored at −80°C until RNA extraction. Finally, blood from 81 additional fish were sampled in order to immediately estimate immune parameters (detailed below). The hepatic and spleen somatic indices were determined as:

(3) $$\begin{array}{l} HSI=\frac{LW}{BW}\times 100 \end{array}$$

and

(4) $$\begin{array}{l} SSI=\frac{SW}{BW}\times 100 \end{array}$$

with BW, the mean body mass (in g wet weight) and LW et SW, the weight of the liver and spleen (in g), respectively.

### 2.5. The Apparent Digestibility Trial

Juveniles, randomly sampled from the same cohort as the previous experiment, were transferred to the experimental INRAE facility of Saint-Pée-sur-Nivelle (France) in order to analyse the apparent digestibility coefficient (ADC). Groups of 15 fish, weighing on average 120 g, were maintained in 60L cylindro-conical tanks equipped with an automatic feces collector (3 tanks per condition), at a constant temperature of 17°C between 392 and 454 dpf. Fish were hand-fed twice a day to visual satiation with either the V or the M diet (MR group not included), enriched with 1% Cr_2_O_3_, as an inert marker. The feces were automatically collected over 62 days and stored daily at −20°C for further biochemical analyses.

Diets, previously reduced to powder, and feces which were previously lyophilized were analyzed. Dry matter content (DM) was measured after drying samples (5 g) at 105°C for 24 h. Protein content was estimated by the Kjeldahl method (Nx6.25, Kjeldahl Nitrogen Analyser 2000, Fison Instruments). The gross energy of the samples was measured after combustion in an adiabatic bomb calorimeter. Starch content was measured according to the methods described by Thivend et al. (1972). Total lipid extraction was performed according to Folch et al. (1957), to assess final lipid content.

The apparent digestibility coefficients (ADC) were calculated as:

(5) $$\begin{array}{l} ADC_{X}=100-\left(100*\frac{X_{feces}}{X_{diet}}*\frac{Cr_{2}O_{3diet}}{Cr_{2}O_{3feces}}\right) \end{array}$$

where X corresponds to protein, lipid, starch, or energy.

### 2.6. Analysis of Plasma Metabolites and Immune Parameters

Plasmatic levels of glucose, triglycerides and cholesterol levels were measured using three commercial kits (Glucose RTU, bioMérieux, Marcy l'Etoile, France; PAP 150, bioMérieux; CHOD-PAP, Sobioda).

Haematocrite (Ht) were determined by the microhaematocrit technique after centrifugation at 12,000 g for 5 min. Whole blood was diluted at 1/200 in a Giemsa solution according to the method of Kekic and Ivanc (Kekic and Ivanc, 1982). White blood cell counts (WBC) were performed with a Thoma's cell haemocytometer. Plasma lysozyme activity was determined using a turbidimetric assay (Grinde et al., 1988) and adapted to microtitration plates, where a bacterial suspension of *Micrococcus lysodeikticus* (Sigma; 1.25 g.L^−1^ in a 0.05 M sodium-phosphate buffer, pH 6.2) and 50 μL of the individual samples were placed in 96 well microtitration plates and the optic density reading was taken at a wavelength of 450 nm every 15 s for 3 min. Lysozyme concentrations for each of the samples were converted to mg mL^−1^ of plasma using the reference curve established with hen egg white lysozyme (Sigma). Determination of the alternative pathway of plasma complement activity was carried out by hemolytic assay with rabbit red blood cells (RRBC, Biomérieux) as described by Yano (1992) and adapted to microtitration plates. Plasma samples, diluted to 1/64 in EGTA-Mg-GVB buffer to prevent natural haemolytic activity, were added in increasing amounts, from 10 to 100 μL well^−1^. The round-bottomed wells were then filled with EGTA-Mg-GVB buffer to a final volume of 100 μL, and a 50 μL of 2% RRBC (Biomérieux) suspension was finally added to all wells. Control values of 0 and 100% haemolysis were obtained using 100 μL EGTA-Mg-GVB buffer and 100 μL haemolysis serum at 1/50 in water, respectively. Samples were incubated for 1 h at 20°C. The microplates were then centrifuged (400 g, 5 min, 4°C). Next, 75 μL of supernatant from each well was transferred with 75 μL of phosphate buffer saline (PBS, Biomérieux) into a 96-well flat-bottom microplate. The absorbance (ACH50) was read in a Labsystems'iEMs analyser at 540 nm and the number of ACH50 units per ml of plasma was determined by reference to 50% of haemolysis.

### 2.7. Molecular Analyses: Liver Transcriptomic Profiles and qPCR

#### 2.7.1. RNA Extraction

Total RNA was extracted from liver (*n* = 6 per treatment), using the TRIzol^®^ reagent method (Invitrogen, Carlsbad, CA, USA), according to the manufacturer's recommendations. The concentration of extracted RNA was analyzed using a spectrophotometer (ND-1000, NanoDrop) by measuring absorbance at 260 nm. The quality of RNAs was checked with Bioanalyzer (Agilent Technologies, Kista, Sweden).

#### 2.7.2. Microarrays, cDNA Labeling, and Hybridization

Liver transciptome profiles of the three isogenic lines were analyzed with microarray technology. Microarray analyses were performed on an Agilent-based microarray platform RBT specific with 8 X 60 K probes per slide, which were previously employed and validated (Callet et al., 2018).

For each sample, 150 ng of total RNA was first amplified by a reverse transcription, using a polyDT T7 primer (denaturation step: 10 min at 65°C, reaction step: 2 h at 40°C, inactivation step: 5 min at 70°C). The obtained cRNA were labeled with Cy3-dye (2 h at 40°C). Excess dye was removed using a RNeasy kit (Qiagen). The level of dye incorporation was evaluated using a spectrophotometer (Nanodrop ND1000, LabTech) (Yield > 0.825 μg cRNA and specific activity > 6pmol of Cy3 per μg of cRNA). 600ng of Cy3-cRNA was then fragmented with a specific buffer (30 min at 60°C). Cy3-cRNA were then hybridized on a sub-array (17 h at 65°C in a microarray hybridization oven (Agilent). Slides were washed and scanned (Agilent DNA Microarray Scanner, Agilent Technologies, Massy, France) using the standard parameters for a gene expression 8x60K oligoarray (3 μm and 20bits). Data were then obtained with the Agilent Feature Extraction software (10.7.1.1).

#### 2.7.3. Strategy to Detect Potential Molecular Markers

The goal of the analysis was to deviate from the traditional studies exploring the effects of plant-based diets, in order to detect potential molecular markers linked to plant-based diet utilization i.e., genes differentially expressed among the three lines which could explain growth differences observed when fish were fed the V diet. First, the transcriptomic profile of isogenic lines were compared amongst one another when fed the V diet (R23h-V vs. AB1h-V; R23h-V vs. A22h-V; AB1h-V vs. A22h-V). However, expression of these genes could vary among lines with no relation to their different abilities to use the V diet, as the three lines display different genotypes. To remove those genes related to genotype, genes which were differentially expressed among lines when fish were fed the M diet *ad libitum* (R23h-M vs. AB1h-M; R23h-M vs. A22h-M; AB1h-M vs. A22h-M) were excluded from further analyses.

For each line comparison, the list of genes obtained were considered as potential molecular markers. Among these genes, some were related to the capacity to grow with a plant-based diet and some were related to difference in feed intake among lines. To differentiate these two types of molecular markers, lines were compared when fed the restricted M diet (R23h-MR vs. AB1h-MR; R23h-MR vs. A22h-MR; AB1h-MR vs. A22h-MR). Genes which were only differentially expressed between lines when fish were fed the V diet were considered as potential molecular markers linked exclusively to plant-based diet utilization (V ∩ (M ∪ MR) ^*C*^). Genes differentially expressed between lines when fish were fed both the V and the restricted M diet were considered as potential molecular markers linked to differences in feed intake ((V ∪ MR) ∩ M ^*C*^).

In a second step, to characterize the potential markers, gene ontology (GO) enrichment analyses were performed using the functional annotation chart tool from the DAVID (Database for Annotation, Visualization and Integrated Discovery) bioinformatics resource, version 6.7 (Huang et al., 2009a,b). Gene ontology (GO) enrichment analyses were carried out on the three lists of potential markers linked to the V diet, and three lists of markers linked to feed intake reduction (Cutoff : *P* < 0.01; Benjamini < 0.1). Swiss Prot identifiers were used as input against all Swiss protein identifiers in the array.

In a last step, to help enrich the biological interpretation, the within line diet effect was assessed for genes previously kept, by calculating the fold change between fish fed the V diet vs. the control M diet *ad libitum* (R23h-V vs. R23h-M; AB1h-V vs. AB1h-M; A22h-V vs. A22h-M) (Cut-off *P* = 0.05).

#### 2.7.4. qPCR

qPCRs were performed on genes found differentially expressed thanks to the microarray analysis, in order to confirm the results. Those genes were involved in metabolism (carbohydrate, lipid, cholesterol, amino acid) and immunity.

One μg of total RNA, per condition, was reverse-transcribed to cDNA with SuperScript III RNase H reverse transcriptase (Invitrogen, Carlsbad, CA, USA) using random dT Primers (*N* = 6/condition). qPCRs were performed using the Roche Lightcycler 480 system (Roche Diagnostics, Neuilly-sur-Seine, France). Two μl of diluted cDNA were mixed with 3 μl of Light cycler 480 SYBR^®^ Green I Master mix and diluted to obtain a final volume of 6 μl. Forward and reverse primers were used at a final concentration of 400 nM. Thermal cycling was initiated with an incubation at 95°C for 10 min. Forty five steps of PCR were performed, each one consisting of heating at 95°C for 15 s for denaturing, and at 60°C for 10 s for annealing and a third extension step at 72°C for 15 s. Melting curves were systematically monitored (with a gradient of 0.5°C/10 s from 55°C to 94°C). Primer sequences, along with their efficiencies (ranged between 1.8 and 2.2) are presented in the Additional File 1 in Supplementary Material.

### 2.8. Statistical Analysis

Statistical analyses were performed using the R-software (version 3.2.5). Mean body mass were log transformed to meet the normality and homogeneity of residuals. Two-way analyses of variance (ANOVA) were performed to assess the effects of the dietary treatment, the genotype and the genotype × dietary treatment interaction (cut-off *P* = 0.05). Differences between conditions were assessed with Tukey's range tests (cut-off *P* = 0.05).

Data from the microarray analysis were transformed with a logarithmic transformation, scale-normalized and the quality of each sample was controlled using the ArrayQualityMetrics package (Kauffmann et al., 2009) from the R-software. Data were analyzed using the package Limma (Smyth, 2005) and for each comparison Limma *t*-tests were performed, with a correction for multiple tests (*P*-value cut-off = 0.05 after a Benjamini-Hochberg correction).

Concerning qPCR data, assays were carried out according to the MIQE (Minimum Information for Publication of Quantitative Real-Time PCR Experiments) standards (Bustin et al., 2009). The relative expression levels were calculated with a mathematical method based on the real-time PCR efficiencies, using a geometric mean of two reference genes (*18S* and *eef1a*) for the normalization (Pfaffl, 2001). Gene expression data were transformed with a logarithmic transformation. The normality and homogeneity of variance of residuals were tested with the Shapiro-Wilk's and Bartlett tests, respectively. Pearson's correlations were calculated to quantify relationships between microarray and qPCR data.

### 2.9. Ethic Statement

All experiments were conducted in the INRAE experimental facilities (Peima facilities, Sizun, France and UMR NuMEA, St-Pée-sur-Nivelle, France), which are authorized to conduct experiments on animals through a permit granted by the French veterinary service (B 29-277-02 and A 64-495-1). The experiments were conducted in strict accordance with EU legal frameworks related to the protection of animals used for scientific research (Directive 2010/63/EU) and in accordance with the National Guidelines for Animal Care of the French Ministry of Research (decree n°2013-118, February 1st, 2013). The scientists in charge of the experiments received training and personal authorization (n°B64 10 003 and A29 102). In agreement with the “Comité d'Ethique Aquitaine Poissons Oiseaux”(C2EA-73) and “Comité d'Ethique Finisterien en Expérimentation Animale” (C2EA-74) ethics committees, the present experiment did not require additional ethics approval since it only involved standard rearing practices and all diets used in the experiment were formulated to include all of the rainbow trout's nutritional requirements [The National Research Council, NRC]. During the experiment, fish were monitored daily. If any clinical symptoms (i.e., morphological abnormality, restlessness or uncoordinated movements) were observed, fish were sedated by immersion in 2% benzocaine solution and then euthanized by immersion in a 6% benzocaine solution (anesthetic overdose) over a period of 3 min.

## 3. Results

### 3.1. Fish Performance

#### 3.1.1. Effect of the V Diet and Effect of the Restriction

**Figure 1:**
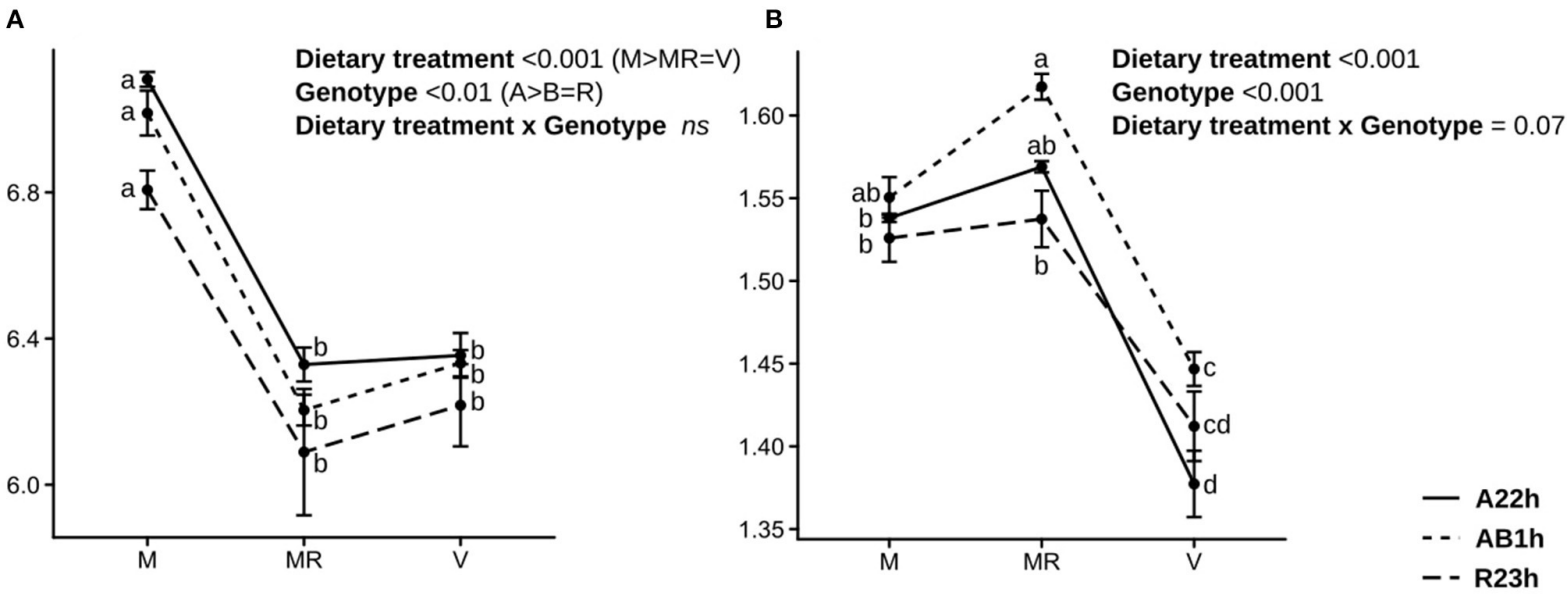
Mean feed intake and feed efficiency. **(A)** Mean feed intake (in g/g metabolic BW/day) and **(B)** mean feed efficiency (means ± standard error) estimated during the 52-day trial for the three isogenic lines (R23h, AB1h, and A22h) fed either the control marine resources-based diet *ad libitum* (M) or restricted (MR) or the complete plant-based diet (V) (*n* = 3). Data were analyzed using a two-way ANOVA to assess the effects of the dietary treatment, the genotype and the genotype × dietary treatment interaction, followed by a *post-hoc* Tukey test in case of significant interaction (different letters mean significant differences between groups).

On average, fish fed the V diet were 26.5% smaller than fish fed the M diet *ad libitum* and fish fed the restricted M diet were 28.0% smaller than fish fed the M diet *ad libitum* (Table 3). Fish fed the V diet had a lower survival rate than those fed the restricted M diet (−31.4%, *P* < 0.001), and those fed the M diet *ad libitum* (−38.5%, *P* < 0.001) (Table 3). Fish fed the V diet also had a 9.7% lower feed intake than those fed the M diet *ad libitum*, and were 9.4% less efficient than the ones fed the M diet, restricted or *ad libitum* (Figure 1). Protein digestibility of the V diet was higher than the M diet whereas the opposite was found for lipid and starch digestibilities (Table 4). Fish fed the V diet had, on average, a 54.6% lower glycemia (due to a lower starch content in the V diet) and a 45.0% lower cholesterol level than fish fed the restricted M diet despite a comparable feed intake (*P* < 0.001 and, *P*-values < 0.001, respectively), and those fed the M diet *ad libitum* (−60.0%, *P* < 0.001 and −44.7%, *P* < 0.001, respectively) (Table 5). After the 5-month-trial, fish fed the V diet had a lower HSI (−51.0%, *P* < 0.001) and a higher SSI than fish fed the M diet, restricted or *ad libitum* (+36.7%, *P* < 0.001, and +80.7%, *P* < 0.001, respectively) (Table 6). Finally, fish fed the V diet had a higher hematrocrite than fish fed the M diet *ad libitum* (+6.8%, *P* < 0.05) and a higher complement activity (+15.0%, *P* < 0.05), but lower lysozyme activities (−37.2%, *P* < 0.05) (Table 6).

**Table 3:** Final body mass (g) and survival rate (%) (means±SEM) of the three isogenic lines (R23h, AB1h, and A22h) when fed either the control marine resources-based diet *ad libitum* (M), the restricted resources-based diet (MR) or the complete plant-based diet (V).

**Dietary treatment**	**Genotype**	**Body mass**	**Survival**
M	R23h	18.3 ± 0.2^*a*^	89.3 ± 3.0
	AB1h	17.1 ± 0.3^*a*^	93.0 ± 1.7
	A22h	17.9 ± 0.0^*a*^	93.6 ± 1.2
MR	R23h	12.9 ± 0.3^*cd*^	79.4 ± 6.3
	AB1h	13.2 ± 0.1^*bc*^	83.3 ± 6.2
	A22h	12.3 ± 0.0^*de*^	85.9 ± 7.9
V	R23h	13.6 ± 0.4^*bc*^	60.8 ± 7.5
	AB1h	14.0 ± 0.1^*b*^	62.0 ± 2.7
	A22h	11.5 ± 0.0^*e*^	46.8 ± 3.8
**STATISTICAL ANALYSIS**			
**Dietary treatment**		** <0.001**	** <0.001** (M < MR < V)
**Genotype**		** <0.001**	*ns*
**Dietary treatment × Genotype**		** <0.001**	*ns*

*Two-way analysis of variance, followed by a Tukey post-hoc test were carried out in order to assess effects of the dietary treatment and the genotype. Different letters mean significance between groups (Cut-off P < 0.05). Significant differences are in bold*.

**Table 4:** Coefficients of digestibility (means ± SEM) of the three isogenic lines (R23h, AB1h, and A22h) fed either the control marine resources-based diet *ad libitum* (M) or the complete plant-based diet (V).

**Dietary treatment**	**Genotype**	**Protein**	**Lipid**	**Starch**	**Energy**
M	R23h	90.5 ± 0.2	96.4 ± 0.3^*b*^	98.9 ± 0.0^*b*^	90.2 ± 0.5^*b*^
	AB1h	91.4 ± 0.2	97.9 ± 0.1^*a*^	99.5 ± 0.0^*a*^	92.1 ± 0.1^*b*^
	A22h	91.6 ± 0.1	97.4 ± 0.0^*a*^	99.4 ± 0.0^*a*^	91.9 ± 0.1^*b*^
V	R23h	96.8 ± 0.3	95.6 ± 0.2^*b*^	92.8 ± 0.4^*d*^	89.1 ± 0.3^*b*^
	AB1h	97.1 ± 0.0	96.0 ± 0.0^*b*^	95.7 ± 0.3^*c*^	89.2 ± 0.1^*b*^
	A22h	97.2 ± 0.0	95.8 ± 0.0^*b*^	96.6 ± 0.0^*c*^	89.8 ± 0.4^*b*^
**STATISTICAL ANALYSIS**					
**Dietary treatment**		**<0.00**1 **(V>M)**	** <0.001**	** <0.001**	** <0.001**
**Genotype**		** <0.05** **(AB1h** **=** **A22h** **>** **R23h)**	** <0.001**	** <0.001**	** <0.01**
**Dietary treatment × Genotype**		*ns*	** <0.00**1	** <0.01**	** <0.05**

*Two-way analysis of variance, followed by a Tukey post-hoc test were carried out in order to assess effects of the dietary treatment and the genotype. Different letters mean significance between groups (Cut-off P < 0.05). Significant differences are in bold*.

**Table 5:** Plasma metabolites (means ± SEM) of the three isogenic lines (R23h, AB1h, A22h) when fed either the control marine resources-based diet *ad libitum* (M), the restricted M diet (MR) or the complete plant-based diet (V).

**Dietary treatment**	**Genotype**	**Glycemia**	**Triglycerides**	**Cholesterol**
M	R23h	1.9 ± 0.1^*b*^	3.1 ± 0.3	3.4 ± 0.1^*abc*^
	AB1h	2.6 ± 0.1^*a*^	4.1 ± 0.3	4.0 ± 0.1^*a*^
	A22h	1.8 ± 0.1^*b*^	3.4 ± 0.2	3.3 ± 0.1^*bc*^
MR	R23h	1.9 ± 0.1^*ab*^	3.8 ± 0.2	3.8 ± 0.1^*ab*^
	AB1h	2.0 ± 1.2^*ab*^	4.3 ± 0.2	4.0 ± 0.2^*a*^
	A22h	1.6 ± 0.2^*bc*^	3.2 ± 0.4	3.1 ± 0.1^*c*^
V	R23h	0.8 ± 0.0^*d*^	3.1 ± 0.2	2.0 ± 0.1^*d*^
	AB1h	0.8 ± 0.0^*d*^	4.6 ± 0.3	2.2 ± 0.1^*d*^
	A22h	0.9 ± 0.1^*cd*^	2.9 ± 0.4	1.8 ± 0.1^*d*^
**STATISTICAL ANALYSIS**				
**Dietary treatment**		** <0.001**	*ns*	** <0.001**
**Genotype**		** <0.05** (AB1h > R23h = A22h)	** <0.001**	** <0.001**
**Dietary treatment × Genotype**		** <0.05**	*ns*	** <0.01**

*Two-way analysis of variance, followed by a Tukey post-hoc test were carried out in order to assess effects of the dietary treatment and the genotype. Different letters mean significance between groups (Cut-off P < 0.05). Significant differences are in bold*.

**Table 6:** Immune parameters (means ± SEM) of the three isogenic lines (R23h, AB1h, A22h) when fed either the control marine resources-based diet *ad libitum* (M), the restricted M diet (MR) or the complete plant-based diet (V).

**Dietary treatment**	**Genotype**	**HSI (%)**	**SSI (%)**	**Hematocrites**	**Complement**	**Lysozyme**
M	R23h	2.3 ± 0.2^*cd*^	0.11 ± 0.01	38.4 ± 1.5	10.4 ± 0.6	14.9 ± 4.0
	AB1h	4.6 ± 0.4^*a*^	0.07 ± 0.00	40.3 ± 1.1	10.6 ± 0.8	18.6 ± 3.3
	A22h	3.3 ± 0.1^*b*^	0.07 ± 0.01	43.0 ± 1.7	9.2 ± 0.4	17.3 ± 3.5
MR	R23h	2.4 ± 0.1^*cd*^	0.08 ± 0.01	38.9 ± 1.4	10.8 ± 0.4	12.2 ± 2.6
	AB1h	3.8 ± 0.2^*b*^	0.05 ± 0.01	42.8 ± 1.4	10.6 ± 0.6	16.0 ± 2.8
	A22h	3.0 ± 0.2^*bc*^	0.06 ± 0.01	41.2 ± 0.3	9.1 ± 0.6	10.5 ± 1.5
V	R23h	1.4 ± 0.1^*e*^	0.012 ± 0.01	43.7 ± 0.9	12.0 ± 0.9	9.1 ± 0.9
	AB1h	1.6 ± 0.0^*de*^	0.012 ± 0.01	42.3 ± 1.1	10.9 ± 0.8	10.3 ± 2.4
	A22h	1.7 ± 0.1^*de*^	0.011 ± 0.01	44.0 ± 1.2	12.3 ± 0.7	12.6 ± 1.9
**STATISTICAL ANALYSIS**						
**Dietary treatment**		** <0.001**	** <0.001** (V>M>MR)	** <0.05**	** <0.001** (V>M=MR)	** <0.01** (M ≤ MR ≤ V)
**Genotype**		** <0.001**	** <0.001** (R23h>AB1h=A22h)	*ns*	*ns*	*ns*
**Dietary treatment × Genotype**		** <0.001**	*ns*	*ns*	*ns*	*ns*

*Two-way analysis of variance, followed by a Tukey post-hoc test were carried out in order to assess effects of the dietary treatment and the genotype. Different letters mean significance between groups (Cut-off P < 0.05). Significant differences are in bold*.

#### 3.1.2. Effect of the Genotype and of the Interaction Diet*Genotype

On average, the A22h fish were 6.5% smaller than fish from the other two lines. While there were no significant differences in the final mean body mass of fish fed the M diet *ad libitum* (17.8 ± 0.2 g), the final mean body mass of fish fed the V or the restricted M diet differed significantly. When fed the V diet, AB1h and R23h fish were 7.3% larger than A22h fish. When fed the restricted M diet, AB1h fish were 7.3% larger than A22h fish.

While no difference in dietary feed intake was observed among the different lines when fed the V diet, AB1h fish had a 5.0% higher feed efficiency than A22h fish. R23h fish had, on average, a 0.8% lower protein ADC than AB1h and A22h fish. Concerning lipid, AB1h fish and A22h fish had higher lipid ADC than R23h fish when fed the M diet only (+1.5%, *P* < 0.01), and no differences were detected when fish were fed the V diet (*P* > 0.05). Concerning starch, AB1h and A22h fish had higher starch ADC than R23h fish when fed the M diet (−0.6%, *P* < 0.001), and also when fed the V diet (−3.9%, *P* < 0.001).

On average, AB1h fish had higher plasma triglyceride levels than R23h and A22h fish (+33.6%, *P* < 0.001). Concerning glycemia, while no differences were detected among lines fed the V and the restricted M diets, AB1h fish had a higher glycemia than R23h and A22h fish when fish were fed the M diet *ad libitum* (+38.8%, *P* < 0.05 and +41.2%, *P* < 0.01, respectively). Concerning the cholesterol level, while no differences were detected among lines when fish were fed the V diet, AB1h fish had a higher glycemia than A22h fish when fish were fed the M diet *ad libitum* (+18.5%, *P* < 0.01).

On average, AB1h fish had a higher HSI than R23h and A22h fish (+64.5%, *P* < 0.001 and +24.9%, *P* < 0.05, respectively), and the R23h fish had a higher SSI than those of the other two lines (+28.8%, *P* < 0.05). While no differences on HSI were detected among lines when fish were fed the V diet, the indices of AB1h fish fed the M diet, restricted or *ad libitum*, were higher than those of A22h fish (M: +39.2%, *P* < 0.001), and R23h fish (M: +97.5%, *P* < 0.001 and MR: +59.7%, *P* < 0.001).

### 3.2. Transcriptome Profiles for the Three Isogenic Lines

**Figure 2:**
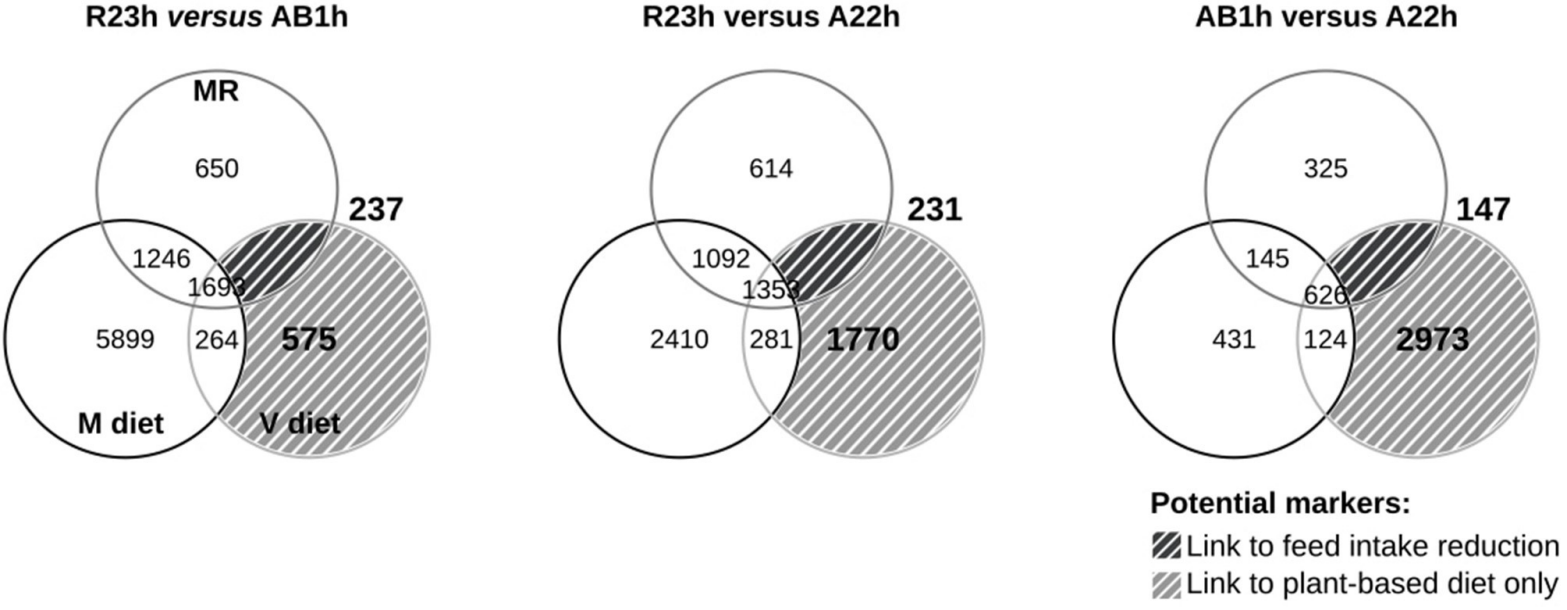
Results of microarray analysis. Venn diagrams showing the number of differentially expressed probes among conditions after limma *t*-test (corrected *P* ≤ 0.05, Benjamini–Hotchberg), when compared the hepatic transcriptomes of the three isogenic lines (R23h, AB1h, A22h) when fed either the control marine resources-based diet *ad libitum* (“M,” black), the restricted M diet (“MR,” dark gray) and when fed the complete plant-based diet (“V,” light gray). Probes differentially expressed between lines only when fish were fed the V diet are considered as potential molecular markers linked to plant-based diet utilization only (V diet ∩ (M diet ∪ MR diet) ^*C*^, light hatched area). Probes differentially expressed between lines when fish were fed both the V diet and the restricted M diet are considered as potential molecular markers linked to feed intake reduction ((V diet ∪ MR diet) ∩ M diet^*C*^, dark hatched area).

The number of probes differentially expressed between the three isogenic lines for each dietary conditions (M, V, and MR) and the number of potential molecular markers linked to the V diet (V diet ∩ (M diet ∪ MR diet)^*C*^) and the feed intake reduction ((V diet ∪ MR diet) ∩ M diet^*C*^) are summarized in Figure 2. Few probes were identified as potential molecular markers linked to the feed intake reduction as only 231, 237, and 145 probes were identified for the R23h vs. AB1h, R23h vs. A22h, and AB1h vs. A22h comparisons, respectively. No gene ontology was identified as enriched in any of the three lists.

Concerning the potential molecular markers linked to the V diet utilization, 575, 1,770, and 2,937 probes were identified for R23h vs. AB1h, R23h vs. A22h and AB1h vs. A22h, respectively. Different GO were obtained for the R23h vs. A22h and the AB1h vs. A22h comparisons (Additional File 2 in Supplementary Material for details), while no gene ontology was identified for the R23h vs. AB1h comparison. These GO were manually classified in different clusters of interest and specific patterns of expression were extracted from the expression data and summarized in Table 7 (Additional File 3 in Supplementary Material for details). Coefficients of correlations obtained between microarray and qPCR data are presented in Table 8. Coefficients of correlations were greater than 0.60 for 18/21 genes tested, demonstrating a high correlation between results from qPCR and from microarray. The lower coefficients of correlations for the 3 genes tested could be explained by the whole genome duplication that occurred in salmonids (Berthelot et al., 2014). Validation of transcriptomic data by qPCR studies is often challenging due to the presence of duplicated and highly similar genes whose transcripts might be differentially regulated (Morais et al., 2011; Lazzarotto et al., 2016). As only 3 genes had lower coefficients of correlation, ranging from 0.46 to 0.60, results obtained by microarray were thus considered as validated by qPCR.

**Table 7:** Patterns of genes expression revealed by the transcriptomic analyses for different pathways of interest.

**Pathways**	**Effect of the genotype**	**Effect of the V diet**		
		**R23h**	**AB1h**	**A22h**
**ENERGY PRODUCTION**				
TCA cycle, respiratory chain	AB1h-R23h>A22h	*ns* (60/76)	*ns* (54/76)	down (62/76)
**LIPID METABOLISM**				
Cholesterol metabolism	AB1h>A22h	up (15/17)	up (15/17)	*ns*/down (15/17)
LC-PUFA biosynthesis	AB1h-R23h>A22h	up (4/6)	up (5/6)	*ns*/down (5/6)
Lipogenesis	AB1h-R23h>A22h	up (4/4)	up (4/4)	up/*ns* (4/4)
Triglycerides biosynthesis	AB1h-R23h>A22h	up (2/4)	up (2/4)	up (3/4)
Prostaglandin biosynthesis	AB1h>A22h	*ns* (2/2)	down (1/2)	down (2/2)
β-oxidation	AB1h-R23h>A22h	*ns* (10/12)	*ns* (10/12)	*ns*/down (10/12)
**PROTEIN CATABOLISM**	AB1h>A22h	*ns* (23/26)	*ns* (22/26)	down (21/26)
**RIBOSOMAL PROTEIN**	R23h-AB1h>A22h	*ns* (32/59)	*ns* (45/59)	down (53/59)
**AA:**				
Catabolic pathways	R23h-AB1h>A22h	*ns*/up (9/11)	*ns*/up (11 /11)	*ns*/down (10/11)
(Tryptophan, L-Leucine, L-Lysine, L-Phénylalanine)				
Transaminases	R23h-AB1h>A22h	*ns* (10/10)	*ns* (7/10)	down (8/10)
Glycine and serine metabolism	R23h-AB1h>A22h	up (8/8)	up (7/8)	*ns* (6/8)
Sulfur amino metabolism	R23h-AB1h>A22h	up (4/9)	up (6/9)	down/*ns* (6/9)
L-arginine biosynthesis	R23h-AB1h>A22h	*ns* (4/5)	*ns* (4/5)	down (4/5)
**CARBOHYDRATE METABOLISM**	No specific pattern	–	–	–
**IMMUNITY**	No specific pattern	–	–	–
(Complement pathways, inflammatory response,				
cytokine activity, T-cell activation and apoptosis)				

*The “Effect of the genotype” displays the differential expression between lines when they were fed the complete plant-based diet (V). The “Effect of the V diet” displays to the effect of the complete plant-based diet for each isogenic line (with “up” = gene was up-regulated when fed the V diet in comparison to the control M diet; “down” = gene was down-regulated when fed the V diet in comparison to the control M diet; “ns” = gene expression was not significantly modified when fed the V diet in comparison to the control M diet., and diet., and “(x/y)” refers to the number of genes included*.

**Table 8:** Correlation between gene expression patterns obtained through real-time PCR and microarray approaches.

**Gene**		**Probes**	**Correlation**	***P*-value**
**LIPID METABOLISM**				
*ptges2*	Prostaglandin E synthase 2	CUST_4249_PI425536763	0.70	5.54E-09
*fasn*	Fatty acid synthase	CUST_6285_PI425536763	0.91	<2.2E-16
*elovl2*	Elongation of long chain fatty acids protein 2	CUST_14393_PI425536763	0.60	4.37E-06
*elovl6*	Elongation of long chain fatty acids protein 6	CUST_15885_PI425536763	0.90	<2.2E-16
*fads6*	Delta 6 Desaturase	CUST_4077_PI425536763	0.46	1.52E-03
*hoad*	3-hydroxyacylA dehydrogenase	TC121766	0.59	5.07E-06
**CHOLESTEROL**				
*hmgcs*	3-hydroxy-3methylglutaryl-CoA synthase	CUST_20666_PI425536763	0.61	1.50E-06
*fdps*	Farnesyl PP synthase	TC120070	0.85	9.36E-16
*lss*	Lanosterol synthase	CUST_28240_PI425536763	0.78	1.38E-11
*mvd*	Diphosphomevalonate decarboxylase	CUST_5335_PI425536763	0.74	4.41E-10
*ebp*	3-beta-hydroxysteroid-Delta(8),Delta(7)-isomerase	TC95915	0.73	1.06E-09
*cyp51a1*	Lanosterol 14-alpha demethylase	TC121294	0.76	4.07E-11
*tm7sf2*	Delta(14)-sterol reductase	CUST_16667_PI425536763	0.81	4.48E-13
**AMINO ACID**				
*csad*	Cysteine sulfinic acid decarboxylase	CUST_19922_PI425536763	0.94	<2.2E-16
*fah*	Fumarylacetoacetase	TC97137	0.53	5.08E-05
*sardh*	Sarcosine dehydrogenase, mitochondrial	TC108740	0.71	4.63E-09
**CARBOHYDRATES METABOLISM**				
*g6pcb2a*	Glucose-6-phosphatase b2.a	TC95453	0.89	<2.2E-16
*pck1*	Phosphoenolpyruvate carboxykinase 1	CUST_2832_PI425536763	0.83	<2.2E-16
**IMMUNITY**				
*C3-3*	Complement C3-3	TC119516	0.84	1.37E-14
*cfb2*	Complement factor B-2	TC116037	0.71	3.67E-09
*lcII*	Lysosyme CII	TC127723	0.93	<2.2E-16

*Coefficient correlation were obtained with Pearson tests*.

## 4. Discussion

We first investigated the effect of a total plant-based diet, without consideration for the genotype background. The lower growth of fish fed the complete plant-based diet was first explained by the large decreased in feed intake. The two-fold higher content of glutamic acid in the V diet, which is known to lower the locomotor activity during feeding behaviors in RBT (Hara, 2006), could be one of the main causes for such observations. The lower feed efficiency, which also contributes to the lower growth, seems to be caused by metabolic disruption (altered hepatic transcriptome) rather than by an altered digestibility (only small diminution of starch and lipid digestibilities). We also confirmed that complete plant-based diets highly affect the fish humoral immune parameters (complement and lyzozyme) (Leaver et al., 2008; Panserat et al., 2008; Geay et al., 2011; Tacchi et al., 2012). Although aquafeed formulations have been significantly improved over the years, the combined effects of an exclusively plant-based diet strongly affect fish and thus the total replacement of fish oil and fishmeal by terrestrial plant ingredients in salmonid aquafeed is not yet feasible.

One of the potential ways to further reduce the use of fish oil and fishmeal is to thus take advantage of the genetic variability which exists in the salmonid population. With this objective, we sought to identify the mechanisms implemented by some trout genotypes to grow more efficiently with a complete plant-based diet, as until now, this question has been little studied (Geay et al., 2011; Overturf et al., 2012; Callet et al., 2018).

The originality of this work rests on the use of the three isogenic lines of RBT as fish share the same genotype within each line, probably reducing variability in gene expressions within each isogenic line. As in the short-term study by Callet et al. (2018), these specific features enhanced the capacity to detect differences between genotypes. Moreover, substitution of FM by plant ingredients in salmonids is known to affect fish feed intake (Gomes et al., 1995; Espe et al., 2006; Dupont-Nivet et al., 2009). As the reduction of feed intake also affects hepatic transcriptome (Salem et al., 2007), we applied a new design in order to dissociate possible differences due to change in feed intake from differences due to the capacity of fish to grow with a 100% plant-based diet.

### 4.1. Different Feed Intake Does Not Explain Differences Among Genotypes

Differences in feed intake is the first hypothesis which may explain growth differences amongst our three genotypes as the existence of a variability in feed intake has already been demonstrated in the RBT population (Dupont-Nivet et al., 2009). While on average, R23h fish have the highest body mass, likely due to a higher feed acceptance in comparison to A22h fish 5 weeks after first-feeding (Callet et al., 2018), no significant differences in feed intake were recorded during the 52-day trial during the juveniles stages (Figure 1). These results were confirmed at the molecular level as only a few potential molecular markers linked to feed intake reduction were found and no significant enrichment of gene ontology was recorded. Together, these results indicate that feed intake level does not explain why some genotypes have a better ability to grow with the plant-based diet, during the juvenile stages.

### 4.2. Large Differences in Efficiency Associated With the Use the V Diet, Despite No Major Differences in Nutrient Digestibility

If not explained by differences in feed intake, growth differences amongst genotypes could be explained by differences in the capacity of fish to utilize the plant-based diet. The A22h fish utilized the V diet less efficiently than the AB1h ones, explaining their lower body mass. The reduced feed efficiency in A22h fish could stem either from a reduced ability to digest nutrients or from a reduced ability to modulate their metabolism. Surprisingly, R23h fish had the weakest capacity to digest nutrients, and in particular lipids, when fed the M diet *ad libitum* and protein and starch regardless of the diet used. However, the differences in digestion capacity arose regardless of the diet used and do not significantly affect their feed efficiency or their final body mass. This result suggests that strong digestive capacities are not a key to efficiently use complete plant-based diets at this stage, in RBT.

### 4.3. Transcriptomic Results Show Major Differences in Metabolism

The transcriptomic results show major differences in metabolism amongst the three genotypes, highlighting the differences that these fish have with respect to how they modulate their metabolism.

#### 4.3.1. A22h: Hepatic Transcriptomes Highly Affected by the V Diet

The most striking result is the overall steadiness of gene expression in the R23h and AB1h in comparison to A22h fish when the effect of the complete plant-based diet was analyzed. There was almost no effect on the expression of an important number of genes related to metabolism and energy production (mitochondrial β-oxydation, proteolysis, amino acid metabolism, the TCA cycle, the respiratory chain and the protein metabolism, including ribosomal protein) in response to the V diet in the R23h and AB1h lines, the two lines with the greatest growth when fed the V diet. The A22h fish were, in contrast, highly affected by the V diet and the molecular response observed (down-regulation of gene expression) was partly in accordance with the typical response exhibited by salmonids fed a plant-based diet.

For genes related to energy production (Panserat et al., 2008) and protein synthesis such as ribosomal protein (Panserat et al., 2008), they were over-expressed in R23h and AB1h in comparison to A22h fish when fed the V diet, suggesting a higher production of energy from ATP hydrolysis, probably used for protein synthesis, and thus, a higher protein turnover in the AB1h and R23h lines. These results directly reflect the higher growth in both lines, AB1h and R23h, and are in accordance with the results obtained in two families of sea bass with different growth rates, which were also correlated with a higher protein turnover (Geay et al., 2011).

Surprisingly, genes involved in proteasome activity were also down-regulated by the V diet in A22h fish. Previous studies showed that both transcription and activity of proteasome were increased when salmonids were fed a diet devoid of FO (Leaver et al., 2008; Panserat et al., 2008), a diet devoid of FM (Vilhelmsson et al., 2004), or a diet devoid of both (Panserat et al., 2009). Yet, proteasome activity is negatively correlated with growth (Dobly et al., 2004), and the down-regulation of genes involved in proteasome activity could reflect the negative effect of the V diet in A22h fish growth.

#### 4.3.2. A22h: No Activation of Pathways Involved in Lipid Metabolism by the V Diet

The existence of a genetic variability in the expression of genes related to lipid and cholesterol metabolisms, already observed in Atlantic salmon (Leaver et al., 2011; Morais et al., 2012) and in RBT (Overturf et al., 2013), seems to be linked to a better ability in some trout genotypes to grow efficiently when fed plant-based diets.

While genes involved in LC-PUFA biosynthesis (elongase and desaturase) were up-regulated by the V diet in the two lines with the highest growth, their expression remained stable in A22h. The results obtained in A22h were in disagreement with results typically obtained when FO and FM are replaced by plant ingredients. To counteract the lack of EPA and DHA in plant-based diets, salmonids typically activate this pathway by over-expressing these genes (Jordal et al., 2005; Leaver et al., 2008; Panserat et al., 2008; Morais et al., 2011; Tacchi et al., 2012). LC-PUFA, and in particular EPA and DHA, play essential roles in the cellular membrane structure and fluidity and are precursors of eicosanoids, either pro or anti-inflammatory molecules (Tocher, 2003). The fact that some genotypes lack or have a reduced ability to activate this pathway could thus have large consequences on other aspects of their physiology and may have contributed to their poor performance when fed complete plant-based diets.

Similarly, genes related to cholesterol metabolism were not up-regulated in the A22h fish, as it could have been expected as plant-based diets are lacking cholesterol, usually bring by FM (Liland et al., 2013). Among these genes, *srebp2* was up-regulated by the V diet in fish with the highest growth, AB1h, and to a lesser extent in R23h fish. However, its expression remained unchanged in fish with lower growth (A22h). This transcription factor regulates expression of the genes implicated in the cascade of cholesterol biosynthesis. Its up-regulation in AB1h and R23h, and its stability in A22h, thus explains the changes in the expression of the other genes. The contrasting abilities to activate the cholesterol biosynthesis pathway are in part reflected in the phenotype as the AB1h fish had on average a higher cholesterolemia than fish from the two other lines, irrespective of the diet. Cholesterol plays an important role within the cell and is a precursor of bile acids. Supplementation of cholesterol in salmon typically represses cholesterol biosynthesis, and increases its conversion into bile acids (Kortner et al., 2014). The two genes involved in bile synthesis (*Bile acid-CoA:amino acid N-acyltransferase*), however, revealed opposite results, as they were down-regulated when fed the complete plant-based diet in AB1h fish. It is thus difficult to determine if the activation of cholesterol synthesis improves bile synthesis in AB1h fish when fed the complete plant-based diet.

Simultaneously, genes involved in the pentose phosphate pathway were over-expressed in AB1h and to a lesser extent in R23h in comparison to A22h when fed the V diet. This result is in accordance with previous results obtained in RBT and in Atlantic salmon, in which the expression of genes involved in pentose phosphate pathways were up-regulated when FM were replaced by mixtures of terrestrial plant ingredients (Vilhelmsson et al., 2004; Panserat et al., 2008; Tacchi et al., 2012). The pentose phosphate pathway produces NADPH, required in different lipid pathways mentioned in the previous sections, but also in lipogenesis. Genes involved in the latter, such as *fasn*, were also more expressed in AB1h and R23h. Together, these results demonstrate the higher capacity of the AB1h and R23h fish to stimulate these different pathways when fed the V diet in contrast to A22h fish.

### 4.4. Immunity: Differences in Gene Expression Only

The transcriptomic analysis also revealed a genetic variability in the expression of genes involved in different immunity pathways, linked to their capacity to grow with a diet devoid of FO and FM. Previous studies found similar results in sea bass and RBT (Geay et al., 2011; Overturf et al., 2012). Although no differences in immune parameters were detected among lines, analyses of gene expression may be more sensitive with respect to detecting differences among the fish.

Several factors are known to affect fish immunity when FO and/or FM are substituted by plant ingredients. The presence of ANF in plant-based diets is one of these factors (Krogdahl et al., 2010). However, a contrasting capacity to cope with ANF among genotypes would result in lipid and protein digestion capacity discrepancies, which is not the case in the present experiment. It therefore suggests that the differences observed in the expression of genes involved in the immune system do not stem from a different capacity to handle ANF.

In addition, FA have key roles in immunity (Montero and Izquierdo, 2010). The unbalanced FA profile in plant-based diets is thus suspected to highly affect immunity (Montero and Izquierdo, 2010), by altering FA composition of immune cell and/or eicosanoid production (Petropoulos et al., 2009), key hormones in immune response derived from FA. Transcriptomic analyses revealed that AB1h and R23h fish could have a higher LC-PUFA biosynthesis capacity. Moreover, *prostaglandin E synthase 2 and 3*, involved in the biosynthesis of both pro and anti-inflammatory eicosanoids, were differentially expressed among AB1h and A22h. While these two genes were down-regulated in A22h, in accordance with previous results obtained in sea bass fed a complete plant-based diet (Geay et al., 2011), expression of these two genes remained unchanged by the V diet in AB1h. Differences in fish capacity to cope with an unbalanced FA profile of the plant-based diet could be the main reason for the differences in the expression of genes involved in immunity.

Finally, differences between plant-based diets and marine resources-based diets also include their AA profile. In this study, the two diets differed in their glutamic acid and leucine contents. Although the roles of that two AA play in immunity have not been widely studied in fish, they are known to have important functions such as the regulation of cytokine production and apoptosis in mammals (Andersen et al., 2016). Differences in their capacity to handle an unbalanced profile of AA could also explain differences in the transcriptomic profile of the three genotypes, with respect to genes related to immune response. Further investigations are needed to understand how nutrients, immune components, and genotype interact and how these interactions might impact growth.

## 5. Conclusion

The high number of genes differentially expressed between fish fed a complete plant-based diet, confirmed the benefit of using these three isogenic lines to investigate the divergent capacity of RBT to grow with such diets. This study, along with the previous one by Callet et al. (2018), allowed us to demonstrate the diversity of strategies that might be implemented by genotypes to survive and grow with plant-based diets devoid of marine ingredients, as well as their diversity over the life cycle.

While some genotypes could more rapidly accept plant-based diets at first-feeding, giving them a long-term advantage over other genotypes (R23h in the present experiment) (Callet et al., 2018), feed intake is no longer a discriminating factor in juveniles. Some genotypes are able to cope with plant-based diet challenges by maintaining different pathways of their metabolism such as the LC-PUFA biosynthesis (AB1h in the present experiment). The immunity component, found in the few studies which investigated the interaction between genotype and a plant-based diet, appears to be a key mechanism in the success of some genotypes to grow with such diets. Still, further studies are needed to understand the intricate interactions that exist between genotype, diet and immunity. Finally, although the complete plant-based diet tested here cannot be used in aquaculture, our experiment shows that breeding programs could be an alternative solution to further diminish the use of FO and FM in the production of high trophic level fish, such as salmonids. Of particular interest, we demonstrated here that some genotypes were better able to activate the biosynthesis of ω-3 LC-PUFA, which is especially important for human consumers. Future research will need to consider commercial populations of salmonids, in order to assess the possibility of using genetic variability in future breeding programs.

## Data Availability Statement

The datasets presented in this study can be found in online repositories. The names of the repository/repositories and accession number(s) can be found here: https://www.ncbi.nlm.nih.gov/geo/query/acc.cgi?acc=GSE179672.

## Ethics Statement

The animal study was reviewed and approved by Comite d'Ethique Aquitaine Poissons Oiseaux and Comite d'Ethique Finisterien en Experimentation Animale.

## Author Contributions

FM, MD-N, EQ, IG, and SS-C conceived and designed the experiments. TK, LL, and PA reared the 3 isogenic lines and collected zootechnical data. IG and CB formulated experimental diets. TC, MC, MD, PA, and AS performed the experiments. TC analyzed the data with discussion with FM, MD-N, SS-C, SP, and MD. TC, FM, and MD-N prepared the final manuscript. All authors read and commented on the manuscript.

## Conflict of Interest

The authors declare that the research was conducted in the absence of any commercial or financial relationships that could be construed as a potential conflict of interest.

## Publisher's Note

All claims expressed in this article are solely those of the authors and do not necessarily represent those of their affiliated organizations, or those of the publisher, the editors and the reviewers. Any product that may be evaluated in this article, or claim that may be made by its manufacturer, is not guaranteed or endorsed by the publisher.
