# Supplementary material for: Why Do Some Rainbow Trout Genotypes Grow Better With a Complete Plant-Based Diet? Transcriptomic and Physiological Analyses on Three Isogenic Lines
Source: Front Physiol. 2021 Sep 1;12:732321. doi: 10.3389/fphys.2021.732321 (PMC8440921; doi:10.3389/fphys.2021.732321)
Supplement: Supplementary file 4 [file Presentation_1.PPTX]

## Slide 1
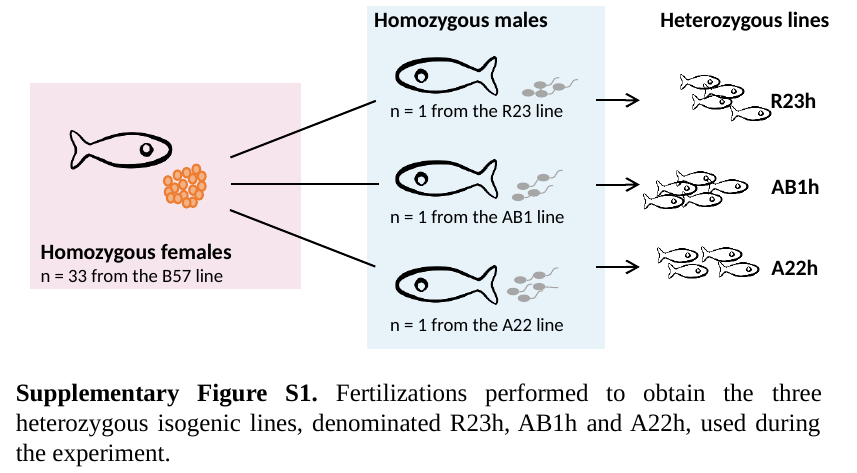

Homozygous males
Heterozygous lines
R23h
n = 1 from the R23 line
AB1h
n = 1 from the AB1 line
Homozygous females
n = 33 from the B57 line
A22h
n = 1 from the A22 line
Supplementary Figure S1. Fertilizations performed to obtain the three heterozygous isogenic lines, denominated R23h, AB1h and A22h, used during the experiment.
